# Incorporating interspecific competition into species-distribution mapping by upward scaling of small-scale model projections to the landscape

**DOI:** 10.1371/journal.pone.0171487

**Published:** 2017-02-16

**Authors:** Mark Baah-Acheamfour, Charles P.-A. Bourque, Fan-Rui Meng, D. Edwin Swift

**Affiliations:** 1 Department of Renewable Resources, University of Alberta, Edmonton, Canada; 2 Faculty of Forestry and Environmental Management, University of New Brunswick, New Brunswick, Canada; 3 Canadian Wood Fibre Centre, Canadian Forest Service-Atlantic Forestry Centre, Natural Resources Canada, Fredericton, New Brunswick, Canada; Aristotle University of Thessaloniki, GREECE

## Abstract

There are a number of overarching questions and debate in the scientific community concerning the importance of biotic interactions in species distribution models at large spatial scales. In this paper, we present a framework for revising the *potential distribution* of tree species native to the Western Ecoregion of Nova Scotia, Canada, by integrating the long-term effects of interspecific competition into an existing abiotic-factor-based definition of potential species distribution (PSD). The PSD model is developed by combining spatially explicit data of individualistic species’ response to normalized incident photosynthetically active radiation, soil water content, and growing degree days. A revised PSD model adds biomass output simulated over a 100-year timeframe with a robust forest gap model and scaled up to the landscape using a forestland classification technique. To demonstrate the method, we applied the calculation to the natural range of 16 target tree species as found in 1,240 provincial forest-inventory plots. The revised PSD model, with the long-term effects of interspecific competition accounted for, predicted that eastern hemlock (*Tsuga canadensis*), American beech (*Fagus grandifolia*), white birch (*Betula papyrifera*), red oak (*Quercus rubra*), sugar maple (*Acer saccharum*), and trembling aspen (*Populus tremuloides*) would experience a significant decline in their original distribution compared with balsam fir (*Abies balsamea*), black spruce (*Picea mariana*), red spruce (*Picea rubens*), red maple (*Acer rubrum* L.), and yellow birch (*Betula alleghaniensis*). True model accuracy improved from 64.2% with original PSD evaluations to 81.7% with revised PSD. Kappa statistics slightly increased from 0.26 (fair) to 0.41 (moderate) for original and revised PSDs, respectively.

## Introduction

Predicting the natural distribution of species across the landscapes has often focused on abiotic-centric species distribution models. These models often ignore biotic interactions and assume that such biotic factors at the scale of the forest stand or patch apparently averaged out, remain constant, or play a nominal role in regulating species distributions at the much larger scale of the landscape [1,2]. Evidence exists, however, that suggests that interaction between species can strongly impact how climate affects geographic range of species [3–7]. Species natural range may shrink in the presence of another species with similar environmental requirements, making predictive errors inevitable if biotic interactions are not incorporated into the species distribution model. Although Woodward and Beerling [8] suggest abiotic-centric species distribution models should be disregarded and replaced by dynamic vegetation models, Bourque and Hassan [9] and Hassan and Bourque [10] suggest there could be a substantial improvement in a model's predictive accuracy if biotic interactions are integrated into existing abiotic-centric species distribution models.

The extension of abiotic-centric species distribution models by incorporating biotic interactions into the modeling framework is often difficult to achieve. Many researchers use modeling approaches that require information that is usually not available for most species or rely on additional model parameterization that obscures aspects of plant behavior [11]. Another concern is that modelers, either consciously or subconsciously, tend to choose permissible parameter values to describe biotic interactions that enhance model performance [12]. Such parameterization usually results in a loss of information and conceals species features that are known to impact their ability to compete with other species. Model prediction of species distribution may well be biased by this parameterization process.

The fingerprint of how biotic interactions, such as interspecific competition, affect species distribution is associated with the direction of forest succession over the long term [13]. In individual-based forest succession models, such as JABOWA [14–16], the differences in species’ physiological tolerances to the various climatic factors determine the intensity of interspecific competition and the direction of forest succession. The roles of climatic factors in regulating species composition and dynamics play out across time, and what is left is a reasonable estimate of the relative competitive ability of different species given different environmental conditions. In a series of research undertakings, Clark et al. [17] suggested that gap models represent competition better than most global dynamic vegetation models (GDVM; e.g., [18]), because competition is modeled at the level of individual plants instead of plant groupings based on functional types.

Results from gap models, scaled up from shifting-gap mosaics to the landscape, could be combined to existing SDMs [19–22]. Such combination is possible for models whose modular form makes the approach adaptable including the potential species distribution (PSD) model by Bourque and Hassan [9]. The final results can provide a rigorous connection between complex mechanistic models of species interaction and species distribution models [23]. However, it remains uncertain whether scaling up results from all types of forest gap models, and incorporating the results into existing abiotic-factor-based species distribution model can help enhance the predictive performance of species distribution model.

In this study, we describe a simple but practical approach to identifying how interspecific competition shapes current large-scale potential distributions of 16 tree species native to the Western Ecoregion of Nova Scotia, Canada. Furthermore, we aim to enhance the PSD modeling approach described in [9–10, 24] to account for long-term interspecific competition among species by including biomass data from forest succession simulated with a robust forest-gap model (i.e., JABOWA-III). Normalized values of differential species performance within forestland types were generated from JABOWA-III simulations of forest succession scaled up to the landscape using a hybrid unsupervised–supervised forestland classification scheme, described in [24]. In contrast to previous studies that investigate the impact of interspecific competition on species distribution at local scales (e.g., [25–26]), our discussion focuses on the more overlooked phenomenon of tree species distribution at regional scales at spatial resolutions suitable for land-management planning (<100 m).

## Materials and methods

### Study area

The Acadian Forest Region of eastern Canada [27] includes the three Canadian Maritime Provinces: Nova Scotia (NS; excluding the Cape Breton Highlands; [28–29]), Prince Edward Island, and all but the northwestern corner of New Brunswick. This investigation uses the Western Ecoregion of NS as primary study area. Ecoregions are ecological land classification units that delineate macroclimatic differences at a provincial scale [30]. The Western Ecoregion extends from Yarmouth to Windsor, including the Halifax peninsula. Geographically, the area is located between 43° 27^'^ to 44° 56^'^ North latitude and 64° 02^'^ to 65° 47^'^ West longitude, with a total land area of 16,904 km^2^, representing about 31% of the total provincial land base. Regional variation in climate is largely influenced by the area’s proximity to the Bay of Fundy in the north and the Atlantic Ocean from the northeast–southwest. The region’s climate is characterized by cold winters and warm springs and summers.

Regional forests are home to about 32 tree species and remain a diverse mix of both conifer and broadleaf species [28, 31]. However, as a demonstration of the procedure, we apply the calculations to 16 target tree species from the 32 common species. These target species are sufficiently abundant to be modeled (>200 observations) and include eight conifer species (i.e., balsam fir (*Abies balsamea* (L.) Mill.), black spruce (*Picea mariana* (Mill.) B.S.P), eastern hemlock (*Tsuga canadensis* (L.) Carr.), eastern larch (*Larix laricina* (Du Roi) K. Koch), eastern white pine (*Pinus strobus* L.), red pine (*Pinus resinosa* Ait.), red spruce (*Picea rubens* Sarg.), white spruce (*Picea glauca* (Moench) Voss)) and eight broadleaf species (i.e., American beech (*Fagus grandifolia* Ehrh.), red maple (*Acer rubrum* L.), red oak (*Quercus rubra* L.), sugar maple (*Acer saccharum* Marsh.), trembling aspen (*Populus tremuloides* Michx.), white ash (*Fraxinus americana* L.), white birch (*Betula papyrifera* Marsh.), and yellow birch (*Betula alleghaniensis* Britton)).

### Data to generate maps of PSD

Data required to generate maps of PSD, include (i) a Digital Elevation Model (DEM) of the Western Ecoregion of Nova Scotia, (ii) precipitation surface, (iii) spatial variability of PAR and SWC generated from the **Lan**dscape **D**istribution of **S**oil moisture, **E**nergy, and **T**emperature (**LanDSET)** model [9, 10], and (iv) remote sensing-based calculations of growing degree days (GDD) [32]. Precipitation data were obtained from 25 Environment Canada climate stations in the Western Ecoregion of Nova Scotia [31]. The DEM was generated from 3-arc second resolution point-data (~70 m at 45° N latitude) acquired from the NASA Shuttle Radar Topography Mission. Net incoming shortwave radiation served as input to the calculation of photosynthetically active radiation (PAR); estimate of net shortwave radiation was generated by the solar radiation-module of LanDSET. The soil water content (SWC) was generated by the soil water balance module in LanDSET; the algorithm includes a decreasing soil moisture availability function (including evapotranspiration, percolation, and surface runoff) and provisions for the accumulations (precipitation, water flow from upslope region).

A GDD map from thermal remote sensing data was developed for the area based on the standard definition of GDD [32], i.e.,

$$\mathrm{GDD}=\sum_{i=1}^{i=n}\max\left(0,\;T_{\mathrm{avg}}-T_{\mathrm{base}}\right),$$ [1]

where T_avg_ is the average daily temperature, T_base_ is a base temperature threshold set at 5°C, and i = 1… *n*, where 1 and *n* represent the start and end day of the growing season. Remote sensing data used in the development of an enhanced GDD-surface (at 30-m resolution, resampled to 70 m) included: (i) Landsat-7 ETM+ surface reflectance data to provide a one-time estimate of EVI at 30-m resolution; (ii) Moderate Resolution Imaging Spectroradiometer (MODIS)-based 8-day composites of surface temperature (at 1-km resolution) and 16-day composites of enhanced vegetation index (EVI; at 250-m resolution) for the April–October period of 2003–2005; (iii) tower-based 30-min emitted infrared (thermal) radiation to estimate surface temperature; and (iv) point estimates of 30-year averages of GDD (1971–2000) from climate stations within the Western Ecoregion for GDD-surface calibration.

### Species-specific response function and PSD calculation

Curves describing the relationships between species occurrence probabilities and abiotic predictor variables of PAR, SWC, and GDD are based on generic functions scaling species response values between 0 and 1, where 0 represents highly unfavorable growing conditions and 1, optimal growing conditions. Species-specific responses to PAR, SWC, and GDD assumed the following forms, respectively:

$$R_{PAR}=c_{1}\cdot \left\{1-\exp\left[-c_{2}\left(nPAR-c_{p}\right)\right]\right\}$$ [2]

where c_1_ is a scaling factor, c_2_ is the slope of the light response curve, c_p_ is the light compensation point or PAR at which the amount of carbon dioxide released in respiration equals the amount used in photosynthesis and the amount of oxygen released in photosynthesis equals the amount used in respiration [Table 1].

**Table 1:** Parameter values for individualistic species-response functions for photosynthetically active radiation (PAR), soil water content (SWC), and growing degree days (GDD) in Eqs (2–8).

**Species**	Shade class^**a**^	nPAR^**b**^			GDD^**c**^		**SWC**^**d**^		
		c_1_	c_2_	c_p_	GDD_min_	GDD_max_	SWC_min_	SWC_max_	Ѱ
Balsam fir	4	1.046	3.290	0.06	563	2011	0.087	0.999	0.50
Black spruce	3	1.125	2.439	0.09	350	2200	0.087	0.999	0.80
Eastern hemlock	5	1.020	4.165	0.03	1300	2900	0.27	0.60	0.50
Eastern larch	1	1.578	1.188	0.15	400	2500	0.20	0.999	0.71
Red pine	1	1.578	1.188	0.15	1400	2300	0.50	0.95	0.70
Red spruce	4	1.046	3.290	0.06	800	2900	0.20	0.80	0.60
White spruce	2	1.259	1.785	0.12	500	2100	0.20	0.90	0.55
Eastern white pine	2	1.259	1.785	0.12	1100	3400	0.20	0.75	0.53
American beech	5	1.020	4.165	0.03	1300	3500	0.18	0.70	0.58
Red maple	3	1.125	2.439	0.09	550	7250	0.01	0.95	0.50
Red oak	2	1.259	1.785	0.12	1525	3878	0.08	0.95	0.75
Sugar maple	4	1.046	3.290	0.06	800	2900	0.58	0.67	0.38
Trembling aspen	1	1.578	1.188	0.15	800	3000	0	0.92	0.75
White ash	2	1.259	1.785	0.12	1275	5600	0.01	0.999	0.66
White birch	1	1.578	1.188	0.15	400	2400	0.55	0.87	0.40
Yellow birch	3	1.125	2.439	0.09	1100	2900	0.085	0.80	0.52

^a^ 1 represents least shade tolerant, and 5 the most shade tolerant

^b^ Values are derived from the literature, e.g., from [16,34], c_1_ is a scaling factor, c_2_ is the slope of the light response curve, and c_p_ is the light compensation point

^c^ GDD_min_ and GDD_max_ are the minimum and maximum tolerance limits of species, based on a review of the scientific literature (e.g., [34]) and references from the internet—the same parameters are used in JABOWA-III

^d^ 0 and 1 represent the soil’s permanent wilting point and field capacity, respectively [33]

ѱ denotes a species functional parameter that defines the shape of the soil-water response curve.

$$R_{SWC}=\max\left[0,\kappa \xi^{\alpha }{\left(1-\xi \right)}^{1/\alpha }\right],$$ [3]

with

$$\xi =\frac{SWC-SWC_{\min}}{SWC_{\max}-SWC_{\min}},$$ [4]

$$\chi =\frac{\psi -SWC_{\min}}{SWC_{\max}-SWC_{\min}},$$ [5]

$$\kappa =\frac{1}{\chi^{\alpha }\cdot {\left(1-\chi \right)}^{1/\alpha }},\;\text{and}$$ [6]

$$\alpha =\sqrt{\frac{\chi }{1-\chi }}$$ [7]

[33], where SWC_min_, SWC_max_, and ѱ (optimal SWC; with SWC_min_ < ѱ < SWC_max_) denote species functional parameters that define the shape of the soil-water response curve.

$$R_{GDD}=\frac{4\left(GDD-GDD_{\min}\right)\cdot \left(GDD_{\max}-GDD\right)}{{\left(GDD_{\max}-GDD_{\min}\right)}^{2}}$$ [8]

[34], where GDD_min_ and GDD_max_ are the minimum and maximum GDDs and represent the northern and southern limits of species tolerance for GDDs. The abiotic-centric PSD (i.e., PSD_original_) was expressed as multiplicative interaction of species environmental response [9], i.e.,

$$PSD_{original}=R_{nPAR}\times R_{SWC}\times R_{GDD}.$$ [9]

The PSD values range between 0–1, where 0 represents unfavorable site conditions (and, thus, potentially low probability of species occurrence), and 1, superior site conditions (and potentially high probability of species occurrence).

#### Incorporating interspecific competition into original PSD

Cumulative effects of simulated interspecific competition over a 100-year simulation period with JABOWA-III provided the best possible estimate of species composition and aboveground productivity at the end of the simulation period. It is this information that we use to construct expressions of species-specific competitive ratings with Eq 10. Before scaling up JABOWA-III simulations of interspecies competition to the level of the landscape, we first classified the area into 12 forestland types (distinct combinations of tree species a particular site can carry) using surfaces of species distribution patterns generated with the abiotic-centric PSD model (i.e., eq. [9–10,35]). A classification of the area was needed to determine the actual range of forest growing conditions present and also reduce the number of JABOWA-III simulations. The methods employed in the classification and validations of the forestland types are more fully described in [24].

We initialized the JABOWA-III model with tree and environmental data from forest inventory plots located in each forestland type; all inventory plot data were in GIS format, courtesy of the Nova Scotia Department of Natural Resources (NS DNR). Individual tree growth, establishment, and mortality were simulated in JABOWA-III taking into account inter-tree competition, and growth-related physical variables such as sunlight, accumulated GDD, and soil water and nutrient content (http://dx.doi.org/10.5061/dryad.tf7f7). As in Eq (9) and LanDSET simulations, mean monthly precipitation and mean monthly temperatures are based on data acquired from Environment Canada climate stations within the study area. Thus, all elements, but the soil conditions and tree-growth-related elements of JABOWA-III are addressed within Eq (9) (abiotic-only model) but at different temporal resolutions. Replicate simulations of the same forest patch (50 replicates) up to 100 years each yielded averaged tree production and composition trajectories. Competitive species ratings was defined by the total aboveground biomass of target species in cohorts consisting of other species, as a proportion of the maximum aboveground biomass of that particular species under optimum growing conditions on a plot. Values of species’ competitive ratings were then rescaled to derive relative competitive ratings ($P_{100}^{k}$) according to the following:

$${P_{100}^{}|}_{l}^{k}=\frac{AGB_{l}^{k}}{\sum AGB_{l}^{k}}\cdot {\left\{\max\left(\frac{AGB_{l}^{k}}{\sum AGB_{l}^{k}}\right)\right\}}^{-1},$$ [10]

where k and l represent functional dependence on species k (whereby k = 1, 2, 3…. m) and forestland type l (l = 1, 2, 3,…., n), respectively. Species-specific competitive ability by forestland type was then used to weigh original PSDs (by way of Eqs (9) and (10)) and give revised PSDs as follows:

$$PSD_{revised}={P_{100}|}_{l}^{k}\times {PSD_{original}|}_{l}^{k}$$ [11]

where $0.0\leq PSD_{l}^{k}\leq 1.0$.

### Model accuracy assessment

The study area has a system of randomly placed forest-inventory plots. The network of plots provides a nearly continuous forest inventory dating back to the 1960s, incorporating field observations of species composition, growth, and mortality. Observed distributions of tree species in over 1,240 forest-inventory plots across the study sites were used to investigate the degree to which original and revised PSD values reflected actual species occurrence. All inventory plot data were also in GIS format, courtesy of NS DNR. For purposes of assessment, one assumption was that raster cells (i.e., 70 m x 70 m) representing the landscape were considered to have the biophysical attributes needed for species occurrence, where original and revised PSD values were greater than 0.25. The analysis summarizes relative frequency of each of the four possible outcomes as follows: (i) predicted vs. observed species occurrence (this outcome demonstrates positive agreement between predicted species occurrence and plot observation); (ii) predicted vs. observed species absence (this outcome demonstrates positive agreement between predicted species absence and plot observation); (iii) predicted occurrence vs. observed species absence (this outcome is indeterminate with respect to model predictions, as species absence from plots may be the result of other forest-forming factors not addressed in the current definition of PSD, e.g., as a result of species migration, disturbance, and forest conversion); and (iv) predicted absence vs. observed species occurrence (the outcome demonstrates potential inaccuracies in modeled biophysical factors and/or associated species environmental response). Analysis of model accuracy was based on two metrics, namely overall accuracy and kappa statistic [36, 37]. The overall accuracy is the proportion of correctly predicted observations of species’ presence and absence, whereas the kappa statistic corrects the overall accuracy of model predictions by the accuracy expected to occur by chance. The kappa statistics range from zero (very poor model accuracy) to one (perfect fit between predictions and observations).

## Results

### Species competitive ratings by forestland type

Simulated values of relative competitive rating ($P_{100}^{k}$) by species and forestland types are summarized in Table 2. During the 100-year successional period, species projected to possess the greatest competitive ability within a given forestland type have $P_{100}^{k}$-values = 1, whereas those expected to be eliminated from the community have $P_{100}^{k}$-values = 0. Final stages of stand development in all forestland types were dominated by relatively shade-tolerant, long-lived species, such as sugar maple, American beech, eastern hemlock, red spruce, and balsam fir, with significant components of eastern white pine, black spruce, and yellow birch. In the absence of large-scale catastrophic disturbance, as is assumed in our study, final species associations in most forestland types resemble a few of the old-growth forest types remaining on the NS mainland [29].

**Table 2:** Value of relative competitive rating representing species’ performance by forestland type.

Forestland type	Relative competitive ratings ($P_{100}^{k}$)															
	bF^1^	bS	eLa	tA	rP	rS	wP	ewP	wA	yB	eH	rM	rO	sM	wB	**Be**
bS	0.82	1.0	0	0	0	0.80	0	0	0	0	0.52	0.35	0	0	0.43	0
Bs-eLa-Rs	1.0	0.3	0	0.2	0	0.3	0.34	0	0	0	0.80	0.23	0	0	0.34	0
bF-bS	0.81	1.0	0.10	0	0	0	0.22	0.36	0.36	0	0.4	0.50	0.18	0.72	0.09	0.14
rS-wP-eH	0.80	0	0	0	0.72	1.0	0	0.96	0	0.16	0.52	0	0.08	0.20	0.12	0.24
bF	1.0	0.08	0	0.07	0	0	0.52	0	0	0	0	0.05	0	0	0.67	0
rS-bF-wP	0.50	0	0	0	0	0	0.23	0.14	0.81	1.0	0.74	0.22	0.41	0.77	0.10	0.36
bS-wS	0.07	0.27	0	0	0.14	0	0.02	0	0	1.0	0.9	0	0	0.33	0	0
eH-rS	0.08	0	0	0.13	0.23	0.15	0	0.02	0	0	1.0	0	0.15	0.32	0.02	0.04
bF-rM	0.23	0	0	0	0	0	0.27	0	0.17	1.0	0	0.50	0.47	0	0.46	0.03
tA-wB-rO	0	0	0.67	1.0	0	0	0	0.78	0	0.72	0.11	0.11	0.56	0.94	0.32	0
rM-wB-rO	0.05	0	0.22	0	0	0	0.02	0	0	1.0	0	0.23	0.35	0	0.02	0
sM-Be-yB	0.44	0	0	0	0.68	0	0.20	0.24	0.23	0.75	0.08	0.32	0.32	1.0	0.08	0.54

^1^Species code: bF = Balsam fir, bS = Black spruce, eLa = Eastern larch, tA = Trembling aspen, rP = red pine, rS = Red spruce, wP = White pine, ewP = Eastern white pine, wA = white ash, yB = Yellow birch, eH = Eastern hemlock, rM = Red maple, rO = Red oak, sM = Sugar maple, wB = White birch, Be = American beech.

### Original vs. revised PSD model

**Fig 1:**
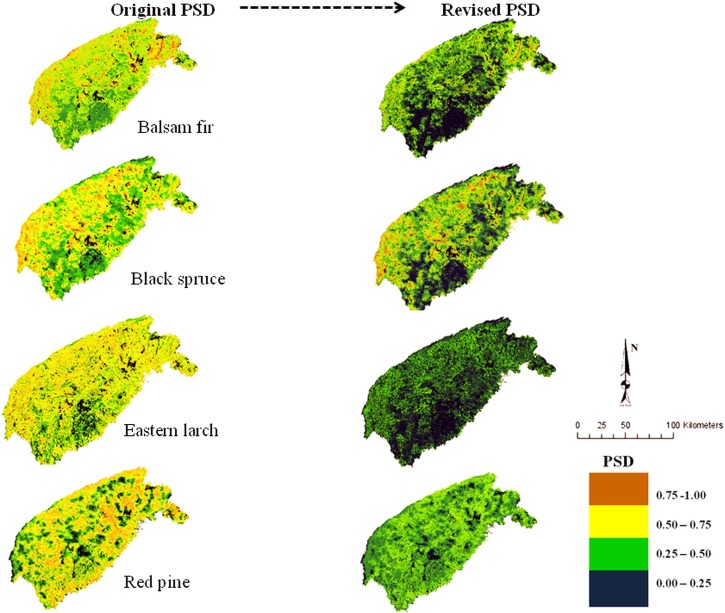
Spatial distribution of modeled PSD surfaces (original vs. revised) for balsam fir, black spruce, eastern larch, and red pine. Original PSD represents species' potential distribution in responses to photosynthetically active radiation (PAR), soil water content (SWC), and growing degree days (GDD); revised PSD (right) indicates distribution in response to PAR, SWC, GDD, and species competitive ability simulated with JABOWA-III forest gap model. Dark blue colors represent least favorable growing conditions and potential absence of species (legend), whereas brown and yellow represent the most favorable conditions and probable presence of the species; green represents intermediate growing conditions and associated species presence.

**Fig 2:**
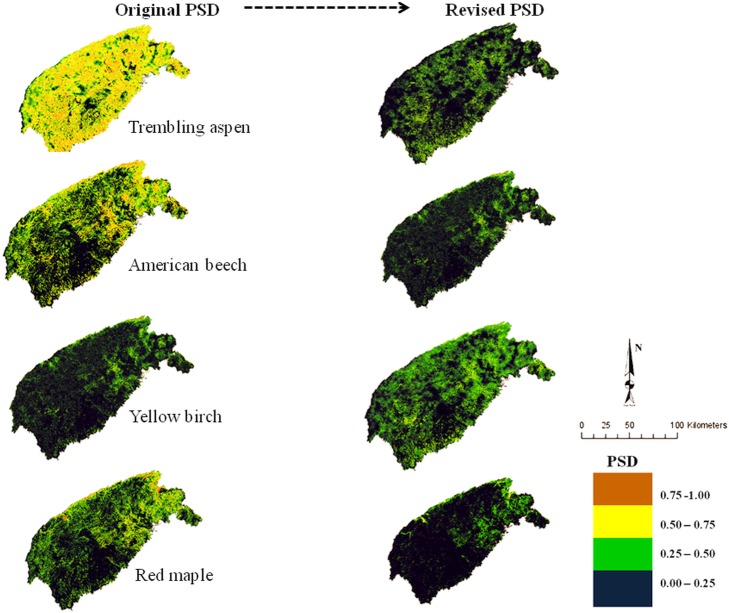
Spatial distribution of modeled PSD surfaces (original vs. revised) for trembling aspen, American beech, yellow birch, and red maple. Original PSD represents species' potential distribution in responses to photosynthetically active radiation (PAR), soil water content (SWC), and growing degree days (GDD); revised PSD (right) indicates distribution in response to PAR, SWC, GDD, and species competitive ability simulated with JABOWA-III forest gap model. Dark blue colors represent least favorable growing conditions and potential absence of species (legend), whereas brown and yellow represent the most favorable conditions and probable presence of the species; green represents intermediate growing conditions and associated species presence.

The original PSD model (i.e., Eq (9)) predicted four conifer (balsam fir, black spruce, eastern larch, and red pine; Fig 1) and two broadleaf (trembling aspen and yellow birch; Fig 2) species to possess the greatest potential to occur across the area. This predicted species occurrence is indicated by the abundance of high-quality sites (represented by yellow and brown colors; accounting for >70% of the land base) in their respective PSD maps.

Four conifer (red pine, red spruce, white spruce, eastern white pine; not shown) and two broadleaf (American beech and red maple; Fig 2) species were predicted to possess intermediate potential to occur in the landscape, as indicated by the abundance moderately high quality sites (i.e., green colors; accounting for >50% of the land base) in the corresponding PSD maps. The original PSD for one conifer (eastern hemlock; not shown) and four broadleaf (red maple, red oak, sugar maple, and white birch; not shown) species revealed that they may not fare as well as other species, because of a narrow response function to the prevailing abiotic factors used in the calculation of potential distribution. This is demonstrated by the abundance of low quality sites (indicated by dark blue color; accounting for >70% of the land base) in their PSD map.

Incorporating a competition model into the original PSD model (by way of Eq (10)) lowered the potential distribution of all species. However, the extent of downgrading varies among species based on individual competitive success within forestland type. The revised PSD map suggested three conifer (balsam fir, black spruce (Fig 1), red spruce (not shown)) and two broadleaf (yellow birch (Fig 2), sugar maple (not shown)) species experienced nominal decline relative to their original distribution. Potential distribution for one conifer (eastern hemlock; Fig 1) and five broadleaf (trembling aspen and American beech, red maple (Fig 2), white birch, red oak (not shown)) species were projected to experience the largest reduction in PSD with the introduction of interspecific competition. For these species, most sites originally described high-to-moderate-quality diminished to low-quality sites, yielding over 86% increase in sites subjected to low levels of interspecific competition. Among all the 16 species studied, eastern larch (Fig 1) and trembling aspen (Fig 2) recorded the greatest reduction in high-quality sites to low-quality sites after adding a competition model to the original PSD model. However, pockets of moderate-quality sites (represented by green colors; Figs 1 and 2) existing across their respective revised PSDs indicate areas where eastern larch and aspen are expected to do well, competitively.

#### Model accuracy assessments

Both the original and revised PSD models (Eqs (9) and (11), respectively) recorded a very high overall accuracy on average (Tables 3 and 4). Addition of interspecific competition improved overall model accuracy from 64.2 to 81.7%. The analysis based on the kappa statistic indicates that there is a greater degree of correspondence between actual species distribution in sample plots and revised PSD, than there is with original PSD (Tables 3 and 4). The kappa statistics increased from 0.26 (fair) with original PSDs to 0.41 (moderate) with revised PSDs.

**Table 3:** Results of an accuracy assessment between original PSD (Eq (9)) values and plot observations. Class limits for the assessment scale are based on Monserud and Leemans [28], namely <0.20 (poor), 0.20–0.40 (fair), 0.40–0.50 (moderate), 0.50–0.70 (good), 0.70–0.80 (very good), and >0.80 (excellent).

		Predicted		Predicted				
		Occurrence		Absence				
**Species**	Plots	vs.		vs.		Overall	Kappa	**Assessment**
	No.	Plot		Plot		Agreement	Statistics	
		Occurrence		Occurrence		(%)		
		Absence		Absence				
Balsam fir	281	64	12	62	143	73.6	0.45	moderate
Black spruce	158	14	4	38	102	73.4	0.29	fair
Eastern larch	76	21	0	36	19	52.6	0.23	fair
Aspen	48	1	0	36	11	25.0	0.01	poor
Red pine	13	1	0	2	10	84.6	0.45	moderate
Red spruce	304	63	42	15	184	81.3	0.56	moderate
White spruce	72	6	2	48	16	30.6	0	poor
White pine	162	42	6	66	48	55.5	0.22	fair
White birch	25	1	1	10	13	56.0	0.02	poor
Yellow birch	53	16	1	21	15	59.5	0.3	fair
Hemlock	47	0	1	1	45	95.5	0.02	poor
Red maple	85	13	4	20	48	71.8	0.34	fair
Red oak	58	0	1	29	28	42.3	0	poor
Sugar maple	27	5	0	4	18	85.2	0.63	good
White ash	136	3	3	48	82	61.4	0.03	poor
Beech	33	8	0	7	18	78.9	0.55	moderate
**Overall**						**64.2**	**0.26**	**fair**

**Table 4:** Results of an accuracy assessment between revised PSD (Eq (11)) values and plot observations. Class limits for the assessment scale are based on Monserud and Leemans [28], namely <0.20 (poor), 0.20–0.40 (fair), 0.40–0.50 (moderate), 0.50–0.70 (good), 0.70–0.80 (very good), and >0.80 (excellent).

		Predicted		Predicted				
		Occurrence		Absence				
**Species**	Plots	vs.		vs.		Overall	Kappa	**Assessment**
	No.	Plot		Plot		Agreement	Statistics	
		Occurrence		Occurrence		(%)		
		Absence		Absence				
Balsam fir	281	64	12	32	204	84.3	0.63	good
Black spruce	158	14	4	22	118	83.5	0.43	moderate
Eastern larch	76	21	0	10	35	84.9	0.69	good
Aspen	48	1	0	14	33	66.7	0.08	poor
Red pine	13	1	0	2	10	84.6	0.44	moderate
Red spruce	304	63	42	5	194	84.5	0.63	good
White spruce	72	6	2	20	46	70.3	0.23	fair
White pine	162	42	6	46	88	71.4	0.42	moderate
White birch	25	1	1	5	19	76.9	0.16	poor
Yellow birch	53	16	1	5	31	88.7	0.75	very good
Hemlock	47	0	1	1	45	95.7	0.02	poor
Red maple	85	13	4	16	52	76.5	0.42	moderate
Red oak	58	0	1	9	48	82.8	0	poor
Sugar maple	27	5	0	2	20	92.6	0.78	very good
White ash	136	3	3	35	94	72.4	0.08	poor
Beech	33	8	0	3	22	90.9	0.78	very good
**Overall**						**81.7**	**0.41**	**moderate**

## Discussion

The original PSD model integrated modeled species-specific response to largely modeled biophysical variables of incident solar radiation (and PAR), SWC, and GDD; the model had been used extensively in eastern Canada to assess tree species habitat suitability [9–10, 38], as well as classify the landscapes into forestland type [24]. However, the impacts of other forest-forming factors beyond the three biophysical variables used in the original definition of PSD were quite large, causing model inaccuracies to be as high as 73% in some areas [9]. We demonstrate a simple procedure for combining results from competition-based forest gap models to the current definition of PSD at the landscape level, without additional parameterization of the original model (i.e., Eq (9)). The critical question is whether scaling up output of gap models, in general, would be useful in explaining plant species distribution.

Results show that accounting for competition effects in the original PSD model provides a better prediction of species distribution, as suggested before [39]. The decline in potential distribution for all tree species in the revised PSD model suggests that tree species cannot be expected to occupy all area of the climatic space that they can tolerate, due to the impact of other factors associated with forest development processes, including intraspecific and interspecific competition [3–7]. We found that mid- to late-successional tree species, such as balsam fir, black spruce, red spruce, yellow birch, and sugar maple, will be least affected by the incorporation of some measure of competition effect into the original PSD model. This effect is confirmed by the nominal decline in moderate- and high-quality sites in their respective revised PSD model. Many of these species are shade tolerant and are unlikely outcompeted for sunlight and thus may occur in many abiotically suitable sites [23,40].

Impacts of adding gap-model-based outputs of competition to tree species distribution were much greater when early successional and shade-intolerant species were considered. For example, eastern larch or trembling aspen, according to the original PSD, can occupy about 86% of the entire area as climatic space that they can tolerate. Incorporating their ability to compete for resources in cohorts consisting of other species reveals that they may not fare as well as initially predicted. As stand-alone species, they grow on a great variety of soil conditions and have the widest distribution of any native tree species in North America [41–43].Although eastern larch and trembling aspen have potential to tolerate a wide range of environmental conditions, realistically, they are present in low abundance and contribute only 3.3% to the total tree volume in the area [44], contrary to what was predicted. Lower real abundance compared with predicted abundance can be attributed in part to low shade tolerance and shorter life spans, rendering them less successful in association with other species over a 100-year period. Apart from low competitive ability, other factors that could cause their actual distribution to be lower include increased fire frequency, intensive harvesting, clearing for agriculture, insects, and diseases [41–44].

We did not use the disturbance option of the JABOWA-III model, other than canopy gap-forming and self-thinning processes. However, there are some residual effects of disturbance captured in the abiotic surfaces, in particular in the expression of temperature and GDDs. Long-term GDD surface is based on 3 years of MODIS images of temperature that we subsequently calibrated with GDDs calculated at climate stations. In general, surface temperatures measured from space or at climate stations, for that matter, possess characteristics representative of the underlying surface. For example, air above forested surfaces, because of high evapotranspiration, tends to be cooler than the air above cutovers, grass surfaces, or airport runways during the day. These temperature differences would clearly become part of the overall expression of GDD, and in that sense would incorporate the effect of disturbance to some extent, introducing a level of noise in the prediction of PSD. Because SWC is based on a water balance calculation and surface temperature (used in the calculation of evapotranspiration, a component of the water balance), the impact of disturbance is also present. However, since SWC is mostly a function of landscape position and flow routing (redistribution of surface and shallow subsurface water; [9]), the effect of disturbance through surface temperature is expected to be considerably smaller. As JABOWA models forest succession based on initializations with tree data from forest inventory plots, the results from JABOWA could also indirectly account for the effects of disturbance. In this study, JABOWA-III takes these disturbances as initial conditions and projects these into the future without additional disturbance.

Despite differences in timescales between Eq (9) and JABOWA, merging information from both models (by way of Eq (9)) does not lead to inconsistencies as they are used to describe a different aspect of PSD, i.e., spatial variation in species habitat (site) suitability vs. a onetime assessment of species aboveground biomass based on 100 years of cumulative effects of interspecies competition and changes in forest composition. However, the JABOWA model also includes a number of abiotic parameters that are not available in the original PSD model. Several site-specific factors addressed in JABOWA (e.g., soil texture, nutrient content, etc.) were not included in the development of the original PSD model at the landscape level. It is implicit that a model with more predictor variables is likely to have better performance [8]. However, over a 100-year simulation period with JABOWA, the role of these abiotic factors in changing species composition plays out across time, and what is left at the end of the simulation period is the relative competitive ability of the different species.

Furthermore, these additional factors were treated as random errors in the original PSD. By including these factors we could have potentially increased model accuracy; however, values for these variables at the landscape level are not readily available. On the other hand, if plot data could have been used to generate these same species’ competitive ratings, naturally the expression of interspecific competition and their accumulation on model output would have been much more exact. Once the above challenges have been addressed, individual-based simulation models are likely to provide outstanding tools for overcoming past limitations and will provide the means to make reliable and robust predictions of the potential distribution of species at the scale of landscapes [6].

## Conclusions

In this paper, we approach the problem of integrating biotic interactions, such as interspecific competition, into an abiotic-centric species distribution model by conducting an upward scaling of results generated with a forest-gap model (i.e., JABOWA-III) from shifting-gap mosaics to the landscape level, by way of Eqs (10) and (11). The results of the revised species distribution analysis indicate that, at the spatial scale of this study, the potential distribution of 16 common trees species in the area is largely a reflection of species’ individualistic response to climatic factors and interspecific competition. Overall, the impact of incorporating some measure of interspecific competition into species’ potential distribution across the landscape was relatively low for late-successional species with high shade tolerance that are dominant across the landscape (e.g., balsam fir, black spruce, red spruce, sugar maple, yellow birch) compared with early successional and shade-intolerant species (e.g., eastern hemlock, American beech, white birch, red oak, red maple, and trembling aspen).

The revised PSD model with interspecific competition (Eq (11)) includes a number of abiotic parameters that are not implicitly addressed in the original PSD model (Eq (9)). Differences between the abiotic-centric and the competition models may, in part, be due to the different input variables used. In future work, the original PSD model will be coupled with those abiotic variables absent in the model (e.g., soil texture, nutrient content).
